# Specialisation events of fungal metacommunities exposed to a persistent organic pollutant are suggestive of augmented pathogenic potential

**DOI:** 10.1186/s40168-018-0589-y

**Published:** 2018-11-22

**Authors:** Celso Martins, Adélia Varela, Céline C. Leclercq, Oscar Núñez, Tomáš Větrovský, Jenny Renaut, Petr Baldrian, Cristina Silva Pereira

**Affiliations:** 1 Instituto de Tecnologia Química e Biológica António Xavier, Universidade Nova de Lisboa (ITQB NOVA), Av. da República, 2780-157 Oeiras, Portugal; 2 Instituto Nacional Investigação Agrária e Veterinária, Av. da República, 2780-157 Oeiras, Portugal; 3 Integrative biology platform, Environmental Research and Technology Platform, Luxembourg Institute of Science and Technology, Belvaux, Luxembourg; 4 Department of Chemical Engineering and Analytical Chemistry, University of Barcelona, Martí i Franquès 1-11, 08028 Barcelona, Spain; 5 Serra Hunter Fellow, Generalitat de Catalunya, Barcelona, Spain; 6 Laboratory of Environmental Microbiology, Institute of Microbiology of the Czech Academy of Sciences, Videnska 1083, 14220 Prague 4, Czech Republic; 7 Institute of Biomedical & Environmental Health Research, School of Science & Sport, University of the West of Scotland, Paisley Campus, PA1 2BE Paisley, UK

## Abstract

**Background:**

The impacts of man-made chemicals, in particular of persistent organic pollutants, are multifactorial as they may affect the integrity of ecosystems, alter biodiversity and have undesirable effects on many organisms. We have previously demonstrated that the belowground mycobiota of forest soils acts as a buffer against the biocide pollutant pentachlorophenol. However, the trade-offs made by mycobiota to mitigate this pollutant remain cryptic.

**Results:**

Herein, we demonstrate using a culture-dependent approach that exposure to pentachlorophenol led to alterations in the composition and functioning of the metacommunity, many of which were not fully alleviated when most of the biocide was degraded. Proteomic and physiological analyses showed that the carbon and nitrogen metabolisms were particularly affected. This dysregulation is possibly linked to the higher pathogenic potential of the metacommunity following exposure to the biocide, supported by the secretion of proteins related to pathogenicity and reduced susceptibility to a fungicide. Our findings provide additional evidence for the silent risks of environmental pollution, particularly as it may favour the development of pathogenic trade-offs in fungi, which may impose serious threats to animals and plant hosts.

**Electronic supplementary material:**

The online version of this article (10.1186/s40168-018-0589-y) contains supplementary material, which is available to authorized users.

## Background

Chemical pollution constitutes a major threat to the sustainability of Earth’s ecosystems; its impacts on biodiversity affect key ecosystem services, such as soil formation and nutrient recycling [1, 2]. Microbes—the unseen majority—are fundamental for the multi-functionality of ecosystems [3], yet progressively hindered by exposure to many disparate chemicals that are spread on a global scale. In particular, chronic exposure to persistent organic pollutants (POPs) released either locally or remotely through long-range atmospheric/oceanic transport is known to dramatically affect the structure, stability and function of microbial communities [4]. Pentachlorophenol (PCP) has a history of use dating back 80 years. Although it was regarded as mostly safe for the first few decades, PCP was eventually included in the Pesticide Action Network’s Dirty Dozen list in 1998 and added to the Treaty of the Stockholm Convention list of banned POPs in 2015 [4], due to its far-reaching toxicity. Its long history of use, coupled with its persistence and ease of transboundary dispersal, has resulted in extensive environmental PCP contamination worldwide [5, 6]. Today, PCP is still detected in human bodily fluids and tissues following exposure in indoor and/or outdoor environments around the world [4].

Recently, we showed the existence of undefined active sources of PCP pollution in the Tabarka district (Tunisia), particularly in soils collected within cork oak forests [4, 7]. The soils were contaminated with PCP levels ranging from 13 to 28 μg/kg of soil. The source and history of the pollution in these soils is unknown [4, 7]. Furthermore, we demonstrated that fungi isolated from these PCP-polluted forest soils can extensively degrade PCP, in theory acting as a buffer against PCP pollution in these habitats [7]. Due to their remarkable catabolic capacities, ubiquitous occurrence and lifestyle [8], saprotrophic fungi possess a peerless ability to degrade harmful chemicals, such as PCP [7, 9–11]. However, regardless of their ability to mitigate pollutants in soils, these activities raise several concerns: How are their communities affected by pollutants at the taxonomic and functional levels? Are there physiological costs underlying the trade-off between PCP degradation and survival?

To address these questions, we have relied on a culture-dependent approach to study the temporal response of a metacommunity of fungi to PCP exposure, uncovering the PCP-derived metabolome, physiological profile, metaproteome and metataxonomy (i.e. stable isotopic probing followed by amplicon sequencing). We show that when confronted with the half maximal effective concentration (EC_50_) of PCP, the metacommunity degraded nearly 70% of the biocide in only 10 days leading, in part, to its mineralisation. Furthermore, we show that PCP exposure altered the taxonomic diversity of the metacommunity, where the loss of some taxa was accompanied by the rise of key PCP-assimilators. It also influenced the proteome within the community; many of the affected proteins were associated with carbohydrate and nitrogen metabolisms. As a final point, PCP pollution was observed to induce functional shifts in the metacommunity suggestive of increased pathogenic potential, which in turn may increase the dispersal of airborne opportunistic pathogens capable of affecting both animal and plant hosts.

## Results and discussion

### The trade-off between PCP degradation and physiological profile

**Fig. 1:**
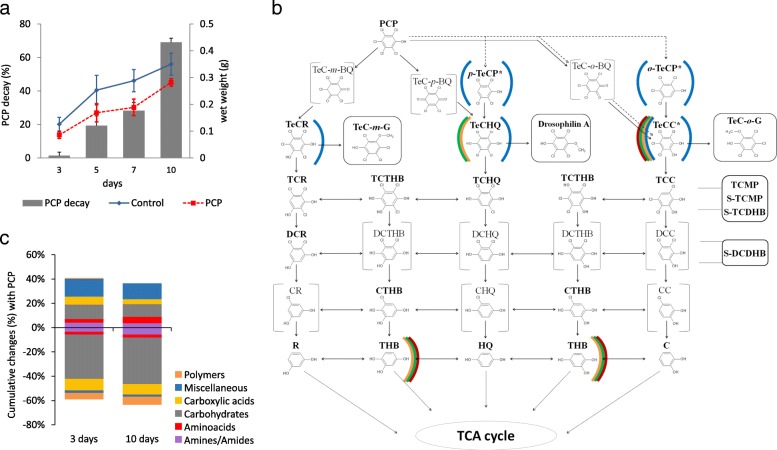
Trade-off between PCP degradation and physiological profile of a metacommunity of fungi: **a** Percentage of PCP degradation throughout the incubation time of the metacommunity exposed to PCP (grey bars). Also shown are the growth curve (fresh weight, FW) for the PCP exposed metacommunity (red line) and the control (blue line). **b** PCP degradation pathway of the metacommunity disclosing the diversity of intra (left brackets) and extracellular (right brackets) PCP-derivatives at the third (blue), fifth (orange), seventh (green) and tenth (red) day of exposure to the biocide. The names of the molecules are depicted as acronyms as follows: TeC (tetrachloro), TC (trichloro), DC (dichloro), P (phenol), BQ (benzoquinone) R (resorcinol), HQ (hydroquinone), C (catechol) and THB (trihydroxybenzene). Additionally, molecules inside boxes indicate conjugations, such as methylations (M). The ortho (*o*), para (*p*) and meta (*m*) isomers are also discriminated whenever needed. Asterisks mark compounds which were identified also at the abiotic control. **c** Utilisation profile of carbon and nitrogen sources by the metacommunity exposed to PCP compared to control conditions (Biolog FF microplates) revealing the cumulative differential utilisation of each substrate category

When exposed to 38 μM PCP—the estimated EC_50_ (Additional file 1: Figure S1)—the metacommunity of fungi ensured the rapid decay of the biocide: PCP decay values ranged from 1.3 ± 2.0 to 69.1 ± 2.4% at the third and tenth day of exposure, respectively (Fig. 1a). Initial modification of PCP by the metacommunity involved its reductive dechlorination, of which the resulting products—tetrachlorophenol isomers (TeCP)—were channelled into the three branches of the PCP degradation pathway: Resorcinol, Hydroquinone and Catechol (Fig. 1b), similar to those reported previously [7, 10, 12]. In fact, on the third day of exposure, tetrachlororesorcinol (TeCR), tetrachlorohydroquinone (TeHQ) and tetrachlorocatechol (TeCC), as well as TeCP, were detected extracellularly (Fig. 1b). Only two compounds were found intracellularly, namely TeCC (throughout the entire incubation period) and TeCHQ (only in the middle of the exposure period, on days 5 and 7). The absence of internalised TeCR (Fig. 1b) suggests that the Resorcinol branch advances at a slower pace than the others, possibly because TeCR formation is preceded exclusively by biotic steps. Trihydroxybenzene (THB) was detected extracellularly at all time subsequent to day 3 (Fig. 1b); its formation may be linked to either branch of the PCP degradation pathway. Detection of THB, together with detection of maleylacetate and 3-oxoadipate (Additional file 1: Table S1, Additional file 2: Dataset S1), directly link the degradation of aromatics to the tricarboxylic acid cycle [12], leading to PCP mineralisation.

To preliminarily uncover the functional costs of PCP degradation within the metacommunity, we analysed the community level physiological profiles (CLPP) at the first and last time points of PCP exposure (Fig. 1a). The biocide did not significantly alter either the functional diversity of the metacommunity (Shannon index, *H′*) or the number of used substrates compared to control conditions. At the final time point, these values ranged from *H′ =* 4.34 (± 0.06) to 4.41 (± 0.02) and *N*_substrates_ = 91.67 (± 0.79) to 94.33 (± 0.58) in the metacommunities exposed or not exposed to PCP, respectively. However, PCP effects on the utilisation of individual substrates were obvious: there was a 60% decrease in the utilisation of carbohydrates and 13% decrease in the utilisation of carboxylic acids; a 35% increase in the utilisation of nitrogen-containing substrates (grouped as miscellaneous) and 14% increase in the utilisation of amino-acids (Fig. 1c, Additional file 1: Table S2, Additional file 3: Dataset S2).

### Scoring PCP assimilators within the metacommunity of fungi confronted with PCP

**Fig. 2:**
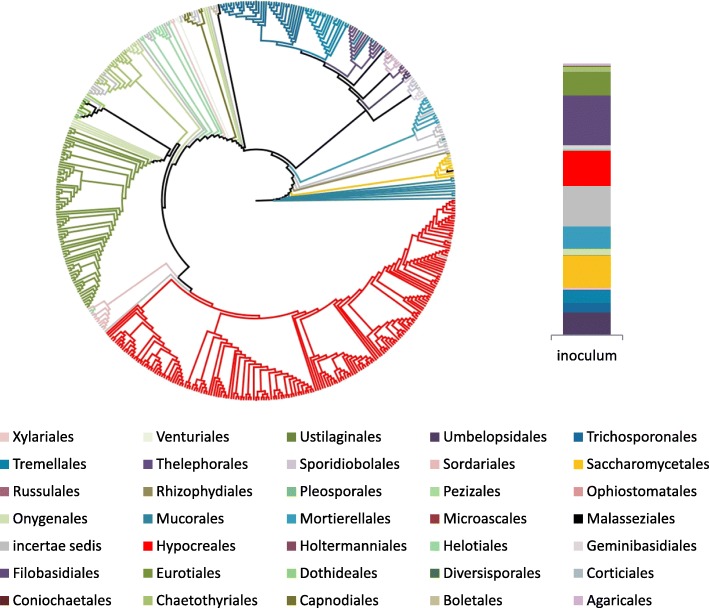
Taxonomic diversity of the metacommunity of fungi in the inoculum. Cladogram based on the ITS2 sequence similarity illustrating the diversity of OTUs identified in the metacommunity inoculum by amplicon sequencing (left) and their relative abundance *per* taxonomic order, comprising also unknown fungi (*incertae sedis*), using the normalised read counts, sub-sampled for the sequencing depth of the Illumina MiSeq run (100000 reads) (right)

**Fig. 3:**
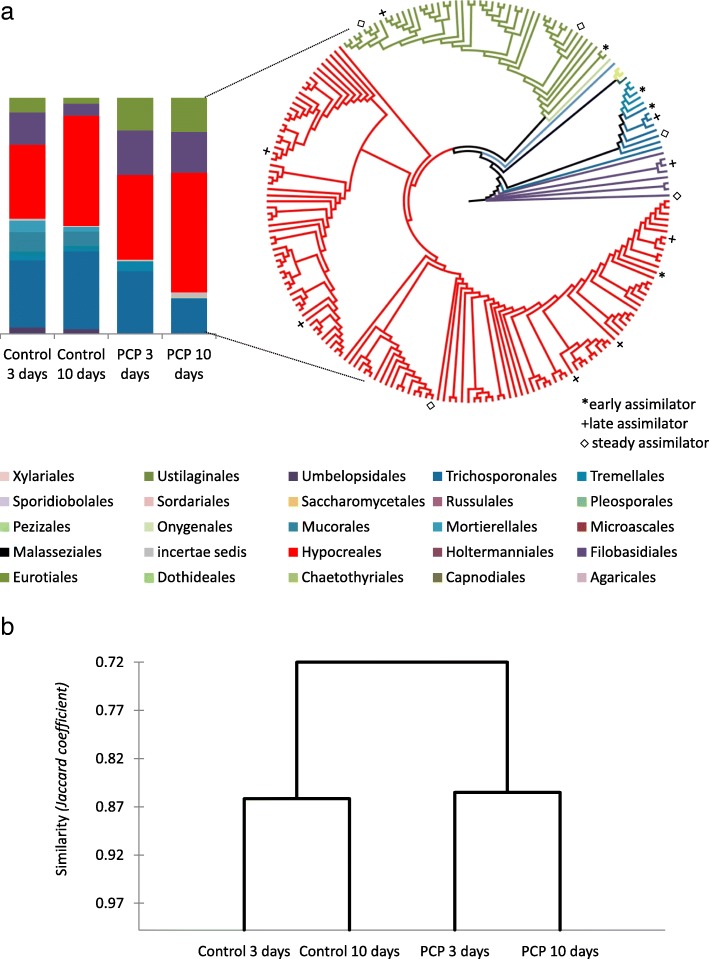
Shifts in the taxonomic diversity of the metacommunity of fungi exposed to PCP. **a** Relative abundances of the identified taxonomic orders in the metacommunity of fungi exposed to PCP on the third and tenth day of incubation, as well as in the corresponding controls (left). The cladogram based on the ITS2 sequence similarity of the metacommunity of fungi on the tenth day of PCP exposure is shown as an example (right), where the marked OTUs correspond to major PCP degraders (see below, Fig. 4). **b** Jaccard-based hierarchical cluster analysis of the taxa diversity of the metacommunity confronted or not with PCP

The observed trade-off between PCP degradation and the community physiological profile may have resulted from shifts in the composition of the metacommunity (culture-dependent approach, see “Materials and methods” section for further details). A total of 398,591 amplicon sequences belonging to fungi were identified using Illumina MiSeq (following trimming based upon quality and size). To achieve a complete fingerprint of the taxonomic diversity, the operational taxonomic units (OTUs) were considered irrespective of relative abundance (excluding singletons) [13, 14]. The inoculum comprised 499 OTUs classified into 36 different orders at distinct relative abundances (Fig. 2). After cultivation for 3 or 10 days, the number of OTUs varied from 228 (21 orders) to 215 (12 orders), and from 178 (16 orders) to 163 (14 orders) in the presence and absence of PCP, respectively (Fig. 3a, Additional file 4: Dataset S3). Cultivation led to the loss of some fungal orders, regardless of PCP levels and occurred even in its absence. In particular, one abundant taxonomic group, Saccharomycetales, which exhibited low OTU sequence diversity, were nearly entirely lost during cultivation (Fig. 2).

Seventy-seven strains were previously isolated by us from the same soil used here as the source of the metacommunity inoculum [7]. As expected, the sequences of the internal transcribed spacer (ITS) region of 34 of these fungal strains matched a few of the OTUs obtained in the present study (defined by their accession number in the CBS-KNAW culture collection, Additional file 4: Dataset S3).

**Fig. 4:**
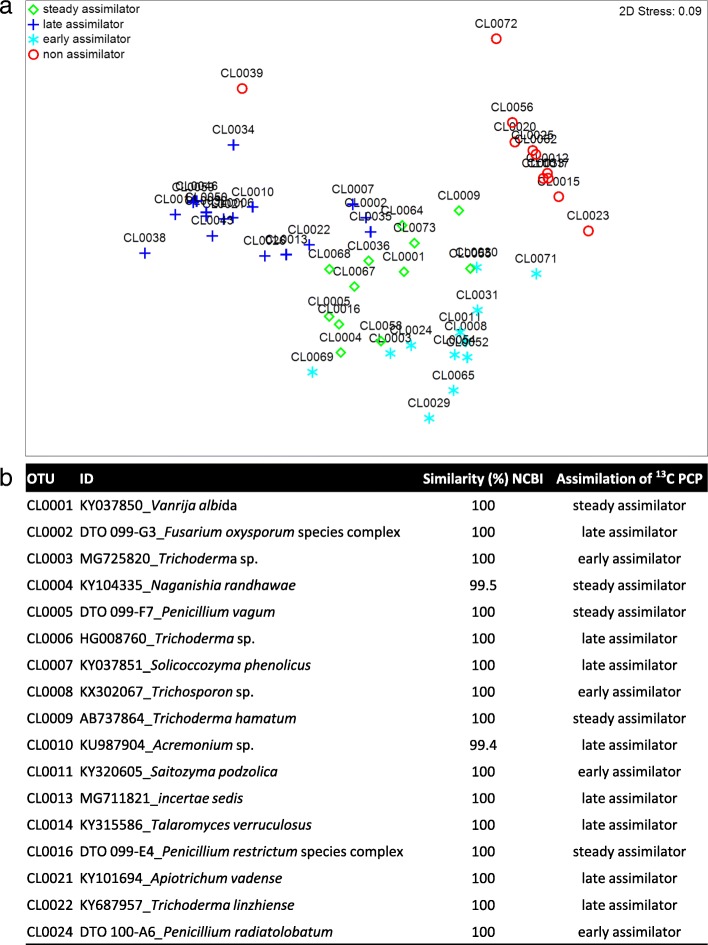
Scoring PCP assimilators within the metacommunity of fungi confronted with PCP: **a** OTUs corresponding to early-, steady-, late- and non- assimilators by the spatial ordination of the normalised OTUs upon multidimensional scaling (MDS) of the constructed Bray-Curtis similarity matrix. **b** The 17 OTUs identified as the most abundant ^13^C-labelled assimilators. The OTUs capable of assimilating ^13^C-labelled were separated in the heavy DNA fraction by isopycnic ultracentrifugation. Major alterations in the abundance of the normalised OTUs were identified using the R-based package *DEseq2*

PCP exposure dramatically altered the share of the most abundant orders: Eurotiales, Filobasidiales and Hypocreales all increased compared to control conditions, while Trichosporonales decreased (Fig. 3a, Additional file 4: Dataset S3). Less abundant taxa that were largely conserved in the control were lost during exposure to the biocide. In particular, PCP decreased the relative abundances of Mortierellales, Mucorales, Umbelopsidales and Tremellales, all of which, except for Tremellales, were absent by the tenth day of exposure (Fig. 3a). PCP affected the taxonomic diversity, i.e. presence versus absence, of the metacommunity (Fig. 3b). To identify the OTUs capable of PCP assimilation, the metacommunity cultures were exposed to stable isotope ^13^C-labelled PCP (Additional file 1: Tables S3 and S4, Additional file 4: Dataset S3). The major ^13^C assimilators were matched to 39 specific OTUs (total reads > 100; Fig. 4a), of which only 17 were particularly abundant (total reads > 1,000; Fig. 4b) largely explaining the multivariate partitioning of data (Fig. 4a). Furthermore, their incorporation of ^13^C-labelled DNA throughout the exposure period suggests distinct roles in the mineralisation of PCP, either as early (^e^), steady (^s^) or late (^l^) assimilators. In this way, the major ^13^C assimilators with regard to the number of OTUs *per* order were Hypocreales (1^s^, 3^l^ and 3^e^), Eurotiales (2^s^, 1^l^ and 1^e^), Trichosporonales (1^s^, 1^l^), Filobasidiales (1^s^, 1^l^) and Tremellales (2^e^) (Fig. 4, Additional file 1: Table S4, flagged in Fig. 3a). Notably, the major assimilators also constituted the dominant taxonomic groups within the metacommunity during cultivation either in the presence (ca. 97%) or absence of PCP (ca. 81% and 86%, on the third and tenth day, respectively). In addition, these taxonomic groups constituted ca. 23% of the total reads in the metacommunity inoculum, suggesting that the biocide has been influencing the mycobiota composition in situ.

### Unveiling the proteome responses of the metacommunity of fungi confronted with PCP

In addition to its effects on the metacommunity composition, PCP also greatly affected the utilisation profiles of carbon- and nitrogen-containing substrates. To verify if such alterations can be detected at the proteome level, changes in the levels of mycelial and extracellular proteins (i.e. secretome) of the metacommunity throughout the PCP exposure (relative to control conditions) were identified. The numbers of mycelial proteins with altered levels (relative to controls; referred to as differential proteins) were 94 and 74 on the third and tenth days of exposure to PCP, respectively. Of these, only 22 proteins were common to both sets. In the secretome, these numbers were 10 and 15, with only 5 common to both time points (Additional files 5 and 6: Datasets S4 and S5).

**Fig. 5:**
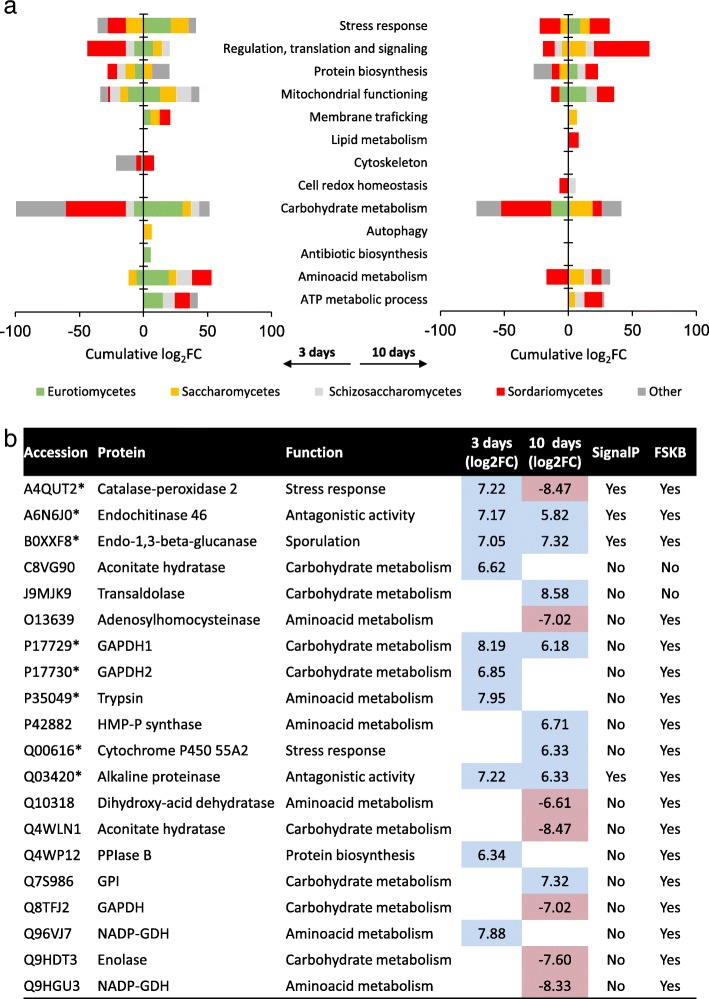
Snapshot of the alterations induced by PCP exposure in the proteome of the metacommunity of fungi compared to control conditions. **a** Changes in the levels of proteins identified in the mycelial proteome at each time point clustered *per* functional category (cumulative fold change, log_2_FC) parsed into the assigned taxonomies (best match at the Uniprot database). **b** Changes in the levels of proteins identified in the secretome, highlighting proteins possibly associated with fungal pathogenic and/or allergenic potentials (*). The accession number (best match in the Uniprot database), short name and functional category, fold change (log_2_FC), signal peptide (SignalP) and presence (or not) at the Fungal Secretome Knowledge Base (FSKB), are indicated. The differential proteins were selected among the identified polypeptides using the R-based package *edgeR*

The snapshot of the effects of PCP on the mycelial metaproteome at each time point is represented by the cumulative fold change (i.e. log_2_FC) of the differential proteins grouped according to gene ontology (GO) functional categories (Fig. 5a, Additional file 1: Table S5). PCP affected many functional categories, of which the most affected (log_2_FC≈|50|) on the third day were carbohydrate metabolism, stress response, mitochondrial functioning, amino acid metabolism and ATP metabolism (Fig. 5a). On the tenth day, the most affected were carbohydrate metabolism, regulation, translation and signalling (Fig. 5a). The most striking difference observed was the major downregulation of carbohydrate metabolism in the presence of PCP throughout the entire exposure period. Metataxonomics discloses the identities at the known taxonomic levels, whereas metaproteomics depends on the best hit of protein sequences available in databases, which is biased toward the best-studied taxa. This may explain the identification of many Saccharomycetes-related differential proteins (viz. Saccharomycetales), of which diversity and abundance were minor factors in the metacommunity; it is possible that these proteins are actually associated with other yet overlooked Ascomycota. Despite this limitation, the majority of the differential proteins were assigned to model fungi related to the dominant taxa observed here (Fig. 3a), which also matched the taxa of the major ^13^C-labelled PCP assimilators (Fig. 4), either Sordariomycetes (viz. Hypocreales) or Eurotiomycetes (viz. Eurotiales) (Fig. 5a). Sordariomycetes become the prominent group at both taxon and protein levels following 10 days of PCP exposure. No proteins related to Basidiomycota were identified, regardless of the fact that Trichosporonales, Filobasidiales and Tremellales were abundant orders in the metacommunity exposed to PCP. On the other hand, many of the proteins exhibiting altered levels were associated with the Schizosaccharomycetes class, which was absent in the metacommunity. The assignment of proteins to a model fungus belonging to this class does not take into consideration its high phylogenetic proximity to Basidomycota [15].

To investigate the biological significance of PCP effects on the mycelial metaproteome, we scrutinised the differential proteins exhibiting the highest log_2_FC. Unsurprisingly, the changes in the levels of many glycolytic enzymes were found to be among the largest changes observed (|7.4| ≤ log_2_FC ≤ |9.7|), either when PCP levels were close to the EC_50_ [aldehyde dehydrogenase; phosphoglycerate kinase; pyruvate kinase; enolases; GAPDH (glyceraldehyde-3-phosphate dehydrogenases)] or threefold lower [GAPDHs; phosphoglycerate kinase; enolases] (Additional file 1: Table S5, Additional file 6: Dataset S5). The levels of aldehyde and alcohol dehydrogenases greatly increased upon PCP exposure (P41751 and P08843, log_2_FC = 8.5 and 7.42). These enzymes have been previously linked to the degradation of many aromatic hydrocarbons, e.g., naphthalene [16]. The involvement of these enzymes in the degradation of PCP (Fig. 1b) is therefore a possibility. Many other enzyme classes have been linked to PCP degradation, e.g., cytochrome P-450 monooxygenases, tyrosinases, reductive dehalogenases and transferases [8]; none of which exhibited increased levels in the mycelial metaproteome following PCP exposure (Additional file 6: Dataset S5). Finally, the ability of the biocide to uncouple oxidative-phosphorylation in mitochondria [4] may be linked to the major dysregulation of some mitochondrial proteins [viz. aconitate hydratase, ATP synthase subunit alpha; citrate synthase; (|7.1| ≤ log_2_FC ≤ |8.5|] (Additional file 1: Table S5, Additional file 6: Dataset S5). The levels of several mycelial proteins associated with the stress response functional category were also greatly increased by exposure to PCP [viz. heat shock protein (HSP) 70 kDa; HSP SSA1; thiamine thiazole synthases; (|7.5| ≤ log_2_FC ≤ |9.2|] (Additional file 1: Table S5). Increase levels of HSP, which act as molecular chaperones, assisting the correct folding of native and stress accumulated misfolded proteins, were reported in *Mucor plumbeus* exposed to PCP [9]. Thiamine thiazole synthase levels increased during adaptation to various stress conditions and are possibly involved in DNA damage tolerance [17].

Regardless of the fact that only a small number of proteins were identified in the differential extracellular metaproteome, it adds important details to the snapshot analysis of the biocide effects (Fig. 5b). Incorporation of data from the extracellular metaproteome adds sporulation and antagonistic activity to the list of affected functional categories (at both time points sampled post PCP exposure). PCP also affected extracellular proteins involved in carbohydrate metabolism, amino acid metabolism, protein biosynthesis and stress response; a similar pattern to that observed in the mycelia (Fig. 5b). Among the extracellular proteins showing increased levels following 10 days of PCP exposure, we found cytochrome P450 (CYP55A2, log_2_FC = 6.3, Fig. 5b). Cytochrome P450 may play a role in the degradation pathway of PCP [8].

### The burden of PCP augments the pathogenic potential of the metacommunity of fungi

When confronted with PCP, the metacommunity adjusted in order to degrade it, leading ultimately to its mineralisation (Figs. 1, 2 and 3). This was achieved via a trade-off resulting in the impairment of many functional categories (Fig. 5) and the increased use of varied nitrogen-containing substrates (Fig. 1c). The ability of fungi to resort to varied nitrogen sources, either to bypass nitrogen starvation or any other conditional metabolic limitation, is critical for the establishment of pathogenicity and considered as a virulence trigger. Taken as examples, the deletion of the gene encoding GAT1, which impaired nitrogen utilisation in *Candida albicans*, lowered its virulence in murine models [18], whereas deletion of AreA/Nit2 gene encoding the transcription factor that controls the expression of genes involved in the transport/catabolism of nitrogen was demonstrated to severely weaken phytopathogenicity in, e.g., *Magnaporthe grisea* and *Fusarium oxysporum* [19].

In line with the hypothesis that PCP may trigger pathogenicity in the metacommunity of fungi, we observed here that nearly half (8 out of 20) of the extracellular proteins that were increased at either sampled time point following PCP exposure have been associated with pathogenic or allergenic potentials, namely GAPDH [20], trypsin [21], catalase-peroxidase [22], alkaline proteinase [23], endochitinase [24], endo-1,3-β-glucanase [25] and cytochrome P450 55A2 (CYP55A2) (Fig. 5b, marked with an asterisk). In addition, throughout incubation in the presence of PCP, the metacommunity greatly increased the pH of the medium, which was unaltered in the controls (Additional file 1: Figure S2). The ability of fungi to change the surrounding pH and to grow at alkaline conditions has also been recognised as a key pathogenicity marker [26]. Finally, the metacommunity grown in media without PCP, after acute treatment with miconazole, showed negligible metabolic activity (absorbance_(570nm)_ = 0.07 ± 0.02), as expected (Additional file 1: Figure S3). On the contrary, and remarkably, following only 10 days of PCP exposure, the capacity of the metacommunity to bypass the effect of the fungicide increased dramatically (absorbance_(570nm)_ = 0.3 ± 0.07), with a concurrent increase in pathogenic potential [27].

## Conclusions

Our results support the hypothesis that fungi pay a high functional cost during exposure to PCP pollution, regardless of superior capacity to degrade the biocide. In a culture-dependent set-up, PCP affected the overall diversity of the metacommunity, in particular it reduced the diversity of the less abundant taxa and promoted also the growth of the most abundant taxa, most of which were capable of assimilating, to some extent, the biocide. Carbohydrate metabolism was critically hindered throughout the entire exposure time, despite the fact that PCP levels were progressively reduced. The metacommunity of fungi circumvented the impacts of the biocide by utilising a variety of nitrogen-containing substrates, which potentially functioned as a virulence trigger. Essentially, PCP exposure greatly reduced the overall susceptibility of the fungal metacommunity to a fungicide and elicited the secretion of proteins that have been found to be associated with pathogenesis.

Atmospheric release of POPs constitutes a silent threat through the chronic contamination of soils on a global scale; yet a fundamental understanding of their impacts is still mostly lacking. The findings of our study extend far beyond the specific issue of PCP pollution. PCP can be considered an archetypal POP since its simple structure—a halogenated aromatic—is found in many POPs that are potentially degraded through biotic pathways that converge on the same pathways used in PCP degradation [8, 11]. Our approach does not intend to directly simulate the natural setting, but it did allow capturing key specialisation events of the metacommunity exposed to the biocide at multiple functional and taxonomic levels; this despite the study’s limits: the loss of some uncultivable taxa and assignment of most proteins sequences to well-known taxa. In this study, the taxa identified as dominant throughout PCP exposure were among the most abundant in the initial soil sample. One hypothesis is that the metacommunity of fungi, which originated from PCP-polluted soils, has been long-suffering the impacts of PCP at both functional and diversity levels. One critical question to be addressed in the near future is how the metacommunity of fungi evolves under chronic PCP exposure conditions, especially as a further imbalance of nitrogen utilisation, with a consequential rise of opportunistic fungal pathogens, should not be ignored. The depletion of nitrogen sources from soils potentially impacts the continuous supply of ecosystem services (e.g., soil fertility and water retention capacity). Therefore, our experimental observations indirectly interrogate if the atmospheric deposition of PCP in cork oak forests may be behind the deterioration observed in these agro-forestry landscapes. Surely, the importance of the link between pollution with PCP (or with other POPs containing similar structural units) and the increase of pathogenic potential in fungi goes way beyond matters of forest sustainability. Annually, fungi kill nearly 2 million people, worldwide [28]. The inhalation of fungal spores, even of non-pathogenic fungi, can lead to devastating invasive infections in vulnerable immunocompromised/suppressed patients of all ages. The stimulation of increased fungal pathogenicity due to POP exposure is not something that can be ignored.

## Materials and methods

### Study design

Microbial communities consist of sub-communities that often contain the same dominant strains, yet contain a distinct composition of the less abundant strains [29]. Conventional culture-dependent assays may favour the development of only a subset of particular sub-communities. To establish a metacommunity of fungi comprising many distinct sub-communities, the community-based cultures were dispersed into many growth containers that were pooled at the end of the experiment, similar to the methodology applied for the establishment of metacommunities composed of several local bacterial communities [29]. Briefly, each biological replicate comprised five 6-well plates (total of 30 wells), each well containing 5 mL of growth medium with or without 38 μM of ^13^C PCP (i.e. EC_50_). The growth media of each biological replicate was mixed with the metacommunity inoculum (ratio of 10:1), then distributed into 30 culture-wells. Cultures were incubated at 30 °C, 90 rpm (triplicates of 30 wells *per* condition) and harvested at the third, fifth, seventh and tenth day of exposure (triplicates). Culture aliquots were used for the physiological profiling. The mycelial and the extracellular fractions were separated using vacuum filtration, the fresh mycelia weight was recorded and both fractions conserved at − 80 °C until further use. The extracellular fractions were used to evaluate the degradation of PCP (viz. PCP residual levels by liquid chromatography and PCP-derived metabolites by mass spectrometry) and the secretome. The intracellular fractions were used to study the community composition (amplicon sequencing), the mycelial proteome and the intracellular PCP-derived metabolites (mass spectrometry). Complementary analyses included measures of the medium pH along cultivation as well as the effect of miconazole on the metabolic activity (MTT reduction assay) of the metacommunity after 10 days of exposure to PCP compared to control conditions (see Additional file 1: Figure S3).

### Chemicals

If not explicitly stated otherwise, chemicals were of analytical grade and purchased from Sigma-Aldrich. All liquid chromatography (LC) and mass spectrometry (MS) solvents were of the highest analytical grade.

### Inoculum of the metacommunity of fungi

The inoculum of the metacommunity of fungi originated from soils sampled inside cork oak forests in Tunisia (E008° 51′ 00.00 N36° 46′ 00.00, Tabarka district, Tunisia) as previously described [7]. In brief, each soil sample is composed by soil collected in each quadrant defined by 1 × 1 m^2^, using a 3-cm-diameter gauge auger at a single depth: 0–20 cm, which was pooled and sieved (< 2 mm). Herein, the three Aîn Hamraia forest soil samples corresponding to distinct forest locations (Additional file 1: Table S7) were carefully combined before use (total soil volume of ca. 2 L). To recover the mycobiota, a soil aliquot (15 g) was immersed (1:10, *w*/*v*) into a solution of 0.1% peptone (*w*/*v*) and 0.1% chloramphenicol (*v*/*v*) (60 min, soft agitation, vacuum cycle every 20 min), then sieved (pore sizes of 500 μm, 210 μm then 100 μm) and finally distributed into 1-mL aliquots that were stored at − 80 °C, as established previously [7].

### Half maximal effective concentration of PCP against the metacommunity of fungi

The half maximal effective concentration (EC_50_) of PCP was determined using 5-mL cultures (6-well plates; 2 plates *per* replicate). Growth media (1% *w*/*v* of glucose in a mineral minimal media [10, 12], MMG) containing 19, 38, 95, 190, 380 or 760 μM of PCP were mixed with the metacommunity inoculum (ratio of 10:1), incubated at 30 °C, 90 rpm for 7 days (triplicates, including negative controls without PCP). Following incubation, 50 μL from each biological replicate (pool of 12 wells) were spread onto MEA and the number of colony forming units (CFUs) monitored daily during 5 days and compared to that of the negative controls (triplicates). To obtain the EC_50_ value, results were adjusted to a logistic regression using the dose effect tool of XL-STAT software version 2009.1.02 (Addinsoft).

### Chemical analyses

PCP was quantified using ultra-performance liquid chromatography (UPLC) as previously described [7]. Chromatographic profiles were acquired at 212 nm and PCP quantification limits were 0.38–56 μM (retention time of 5.9 min). The diversity of PCP-derived metabolites and sub-products in both the extra- and intra-cellular compartments at the third, fifth, seventh or tenth day of exposure to PCP were resolved using ultra high performance liquid chromatography–electrospray–high-resolution mass spectrometry (UHPLC-ESI-HRMS) operated in negative ESI mode using a Q-Exactive Orbitrap MS system (Thermo-Fisher Scientific) as previously described [7, 12, 30]. MS data were processed by ExactFinder™ 2.0 software (Thermo-Fisher Scientific), applying a user target database list and validated, whenever possible, using standard compounds.

### Carbon and nitrogen metabolism

The ability of the metacommunity of fungi to use specific carbon and nitrogen sources was analysed using Biolog FF plates following the manufacturers’ guidelines. The cultures were grown in MMG with or without 38 μM of PCP during 3 or 10 days, as described above, before testing. The plates were incubated at 30 °C and the absorbance of each plate at 490 nm and 750 nm was measured daily for 5 days. Functional diversity (Shannon index, H′) and richness were calculated as previously described [31]. Carbon and nitrogen sources were grouped by category [32]. To reveal functional categories affected on the third or tenth day of PCP exposure compared to controls, the ratios of the increase or decrease of use of each substrate were normalised, and a histogram constructed using XL-STAT software version 2014.5.03 (Addinsoft, France).

### Metataxonomics of the metacommunity-based cultures

The metacommunity diversity on the third and tenth day of cultivation, both in the presence and absence of the stable isotope ^13^C-labelled PCP, was analysed, as well as that of the inoculum, i.e. the metacommunity directly recovered from soils that originated from the cork oak forests. ^13^C-labelled PCP was used to mark OTUs capable of PCP assimilation (see below *Isopycnic ultracentrifugation*).

#### DNA extraction

The frozen mycelia were macerated using a pestle and mortar, then further ground with the aid of an extraction buffer (50 mM of NaH_2_PO_4_, 50 mM NaCl, 500 mM Tris-HCl, 5% SDS, pH 8; 600 μL *per* culture) and glass beads (1 g, equal amounts of 0.5 and 0.1 mm beads) using a TissueLyzer LT Adapter (Qiagen, Germany), for 5 min at top speed. Afterwards, the sample was mixed with a half volume of each: phenol and chloroform containing isoamyl alcohol (24:1; hereafter defined as solution A); shaken for 2 min and centrifuged (5 min, 2,400 *g*) to recover the upper supernatant (i.e. aqueous phase) which was re-extracted with an equal volume of solution A, and recovered as described before. To this mixture, 1/3 volume of 6 M NaCl and 1/10 volume of 10% of cetyl trimethylammonium bromide (CTAB) in 0.7 M of NaCl were added, and the mixture was incubated for 30 min at 65 °C. After cooling to room temperature, an equal volume of solution A was added, shaken and centrifuged (20 min, 1400 *g*) to recover the supernatant. Finally, DNA was precipitated in 2/3 volume of isopropanol and 1/10 volume of acetate solution (3 M) during 20 min at room temperature, and recovered by centrifugation (20 min, 6800 *g*). The DNA pellet was washed with 200 μL of ethanol (70%), recovered by centrifugation as before, air dried for 60 min, eluted in 50 μL of TE buffer (Qiagen, Germany) and finally stored at − 20 °C. Prior to use, the DNA samples were cleaned using the GeneClean Turbo kit for 100–300 kb fragments (MP Biomedicals, USA) following the manufacturer instructions.

#### Isopycnic ultracentrifugation

Isopycnic ultracentrifugation was used to separate the “heavy” (i.e. that incorporated ^13^C) and the “light” DNA fractions, both of which were used to generate amplicon sequencing data (Additional file 4: Dataset S3). The separation of the ^13^C-labelled DNA from the unlabelled DNA was carried out following an established protocol with some modifications [33]. Specifically, the DNA samples were re-suspended in 10 mM ethylenediaminetetraacetic acid (EDTA, final volume of 4 mL), then mixed with 4.7 g of cesium chloride (CsCl) and 10 μL of RedSafe (Chembio Diagnostics, USA) and transferred to 4.7 mL OptiSeal tubes (Beckman Coulter, USA) and centrifuged in an Beckman Optima Max XP, equipped with a TLA-110 rotor, for 40 h, 311,438 *g*, *k*-factor = 21.2, with no break. The light and heavy DNA bands were visualised under a fluorescent light (514 nm) and were recovered by piercing the tube with a syringe. DNA was extracted using a 2:1 butanol/NaCl solution (*v*/*v*, saturated NaCl), washed with ethanol, then eluted in MilliQ water and stored at − 20 °C.

#### Illumina sequencing

For the analysis of fungal community composition, the ITS2 region of fungal rDNA was PCR-amplified in a GeneAmp PCR system 2720 (Applied Biosystems) using barcoded gITS7 and ITS4 primers (gITS7, 5′-GTG ART CAT CGA RTC TTT G-3′; ITS4, 5′-TCC TCC GCT TAT TGA TAT GC-3′) [34] in technical triplicates, including quality controls, as previously described [14]. The quality of the PCR products was monitored using gel electrophoresis. The technical replicates were pooled and sequenced on an Illumina MiSeq system. NGS analysis was performed by the Gene Expression Unit at Instituto Gulbenkian de Ciência (Oeiras, Portugal).

#### Amplicon sequencing data analysis

The amplicon sequencing data were processed using the pipeline SEED 2.1 [13]. Briefly, paired-end reads were joined using FASTQ-join [35]. The ITS2 region was extracted using ITSx1.0.11 [36] before processing. Chimeras were identified using USEARCH 8.1.1861 and deleted. Sequences were clustered using UPARSE implemented within USEARCH [37] at a 97% similarity level. The most abundant sequences were selected for each cluster, and the closest hits were identified using BLASTn against GenBank. Singletons were discarded. The cladograms based on the ITS2 sequence similarity of the identified OTUs were generated using PhyML to illustrate the diversity of the taxonomy within the metacommunity, regardless that the high variability of the ITS2 region does not allow a precise topology. The cladograms were then visualised and edited graphically using FigTree 1.4.3.

### Metaproteomics of the metacommunity-based cultures

#### Extraction of mycelial proteins

Mycelial proteins were extracted using a modified trichloroacetic acid (TCA)/acetone protocol [38]. Briefly, the frozen mycelia (in liquid nitrogen) were ground using a pestle and mortar and homogenised in extraction buffer: 50 mM of Tris-HCl at pH 7.5, 200 mM NaCl, 5 mM EDTA, 0.5% Triton X-100 and EDTA-free EASYpack protease inhibitors (Roche, Switzerland). To facilitate homogenisation and cell rupture, a TissueLyzer LT Adapter (Qiagen, Germany) was used, first 1 g of glass beads (half of each size: 0.5 and 0.1 mm) were added and then two consecutive cycles of 5 min at top speed were applied. Proteins were precipitated in acetone containing 10% (*v*/*v*) trichloroacetic acid (TCA) and 40 mM of dithiothreitol (DTT) (1:10 *w*/*v*) for 1 h at − 20 °C; the pellet recovered by centrifugation at 10,400 *g* for 15 min, washed three times in 10 mL of acetone containing 40 mM of DTT, finally dried under a nitrogen flow and stored at − 80 °C until further analysis.

#### Extracellular protein

The extracellular culture fractions were first concentrated ca. 30-fold using Vivaspin Turbo 15 ultra-filtration systems (Sartorius, Germany). The concentrated samples were mixed with 200 mL of a boiling SDS solution (2% SDS, 40 mM of Tris-base and 60 mM of DTT) and shaken at 99 °C and 350 rpm for 5 min in a Thermomixer (Eppendorf, Germany), then maintained at − 20 °C overnight. Finally, proteins were precipitated in acetone with 60 mM DTT for 1 h at − 20 °C, and recovered by centrifugation (4 °C, 15 min, 21,630 *g*), washed five times with acetone containing 60 mM DTT, finally dried under nitrogen flow and stored at − 80 °C until further analysis.

#### Mass spectrometry analyses of protein extracts

Proteins were recovered from the culture filtrates using denaturing precipitation conditions [9]. Then, 10 μg of protein (quantification performed with RC DC™ protein assay kit, Bio-Rad) was loaded on to a precast gel (Criterion ™ XT precast 1D gel 4–12% Bis-Tris, Bio-Rad) and separated using a short migration. The gel was stained with Instant Blue (Gentaur BVBA, Kampenhout, Belgium), sliced into bands. Proteins were first reduced, then alkylated and de-stained and finally digested using trypsin (sequencing mass grade, Promega). Peptides were extracted, dried and stored at − 20 °C until LC-MS analysis. Peptides were analysed with a nano-HPLC system (NanoLC-2D, Eksigent, Sciex) coupled to a Triple TOF 5600+ mass spectrometer (Sciex, Darmstadt, Germany) operated on positive ESI mode with a Nanospray III source. In detail, after desalting and enrichment on C18 pre-column (C18 PepMap™, 5 μm, 5 mm × 300 μm, Thermo scientific), peptides were separated with a C18 reverse-phase column (C18 PepMap™ 100, 3 μm, 100 Å, 75 μm × 15 cm, Thermo scientific) using a linear binary gradient (A: 0.1% formic acid; B: 80% acetonitrile, 0.1% formic acid) at a flow rate of 300 nL/min. Peptides were eluted from 5 to 55% solvent B over 45 min. Solvent B was then increased to 100% to wash the column before re-equilibrating for 25 min prior to the next injection. The 20 most intense precursors were selected for fragmentation. The CID spectra were processed with Mascot (version 2.4.2) using Mascot Daemon interface (version 2.4.2, Matrix Science, London, UK) by searching against the SwissProt Fungi (31527 sequences) database released on May 2015 and the *Emericella nidulans* (36,970 sequences; 18,794,350 residues) database released on the 13 November 2015. Only the proteins identified with a significance MASCOT-calculated threshold *p* value < 0.05 and at least two significant peptides *per* proteins were accepted.

### Statistical analyses

#### Amplicon sequencing

The amplicon sequencing data attained without any isotopic separation was first treated to assess the overall relative abundances between conditions (incubation with PCP and controls, at the third and tenth day). Descriptive statistics of the OTUs relative abundance and a Jaccard-based hierarchical cluster analysis of their diversity (presence versus absence) were performed using XL-STAT software version 2014.5.03 (Addinsoft, France). The histogram analysis used as weights the normalised number of reads of each OTU (relative abundance) *per* sample (SIP separation not considered), sub-sampled for the depth of the Illumina MiSeq run (100,000 reads).

To study the differential relative abundance of each specific OTU in the light and heavy DNA fractions, we estimated the probability of major fold changes (FC) between the two conditions (negative binomial distribution) as follows: the OTU counts were normalised within each sample and sub-sampled as mentioned above, set to integers and then analysed using the RStudio (version 1.0.153) Bioconductor package DESeq2 [39]. Those presenting differential abundance (normalised counts bigger than 100 reads) between fractions or between the fractions and the controls were classified as ^13^C assimilators. Non-metric multidimensional scaling (NMDS) was used to visualise treatment effects. To build the NMDS, the normalised and sub-sampled counts were standardised within each OTU and then a Bray-Curtis resemblance matrix was built and plotted using a minimum stress of 0.01, using PRIMER 6.1.13 (PRIMER-E, Ltd). The assimilation category of each OTU was used as label.

To test differences at the pH measurements and MTT assays (comparing values obtained after incubation with PCP and controls), Student’s two sample *t* tests were performed after Cohen’s *D* test (to assess the power of variance comparisons, *f* > 0.04) using the tool of XL-STAT software version 2014.5.03 (Addinsoft, France).

#### Metaproteomics

Only the proteins that were present in at least two out of three biological replicates were considered for further analyses. The relative quantification of the proteins has been calculated using the normalised spectral abundance factor (NSAF) [40]. The spectral counts of each mass were normalised and further analysed using the RStudio (version 1.0.153) Bioconductor package edger [41] following generalised linear models [42, 43]. This approach was used to analyse both the mycelial and the extracellular metaproteome. The cumulative log_2_FC for each functional category was plotted using Microsoft Excel, discriminating the contributions of the distinct taxonomic classes.

## Additional files

Additional file 1: Supplementary Information, containing more detailed tables and figures that support the figure panels at the main text. (DOCX 199 kb)

Additional file 2: Mass spectrometry datasets (xls format) on the metabonomics of the metacommunity. (XLSX 16 kb)

Additional file 3: Biolog FF datasets (xls format): normalised datasets of the absorbance of each substrate, disclosing alterations upon exposure to PCP compared to control conditions. (XLSX 28 kb)

Additional file 4: Amplicon sequencing raw count data, including description of the identified OTUs and discrimination of OTUs as 13C-labelled PCP assimilators. (XLSX 104 kb)

Additional file 5: Mass spectrometry datasets on the proteomes of the metacommunity. (XLSX 110 kb)

Additional file 6: List of all the mycelial proteins that underwent alterations after exposure to PCP compared to control conditions. (XLSX 23 kb)
