# Supplementary material for: Specialisation events of fungal metacommunities exposed to a persistent organic pollutant are suggestive of augmented pathogenic potential
Source: Microbiome. 2018 Nov 22;6:208. doi: 10.1186/s40168-018-0589-y (PMC6251201; doi:10.1186/s40168-018-0589-y)
Supplement: Supplementary file 1 — Supplementary Information, containing more detailed tables and figures that support the figure panels at the main text. (DOCX 199 kb) [file 40168_2018_589_MOESM1_ESM.docx]

**Specialisation events of fungal metacommunities exposed to a persistent organic pollutant are suggestive of augmented pathogenic potential**

Celso Martins^1^, Adélia Varela^1,2^, Céline C. Leclercq^3^, Oscar Núñez^4,5^, Tomáš Větrovský^6^, Jenny Renaut^3^, Petr Baldrian^6^, Cristina Silva Pereira^1,7*^

^1^Instituto de Tecnologia Química e Biológica António Xavier, Universidade Nova de Lisboa (ITQB NOVA), Av. da República, 2780-157, Oeiras, Portugal

^2^Instituto Nacional Investigação Agrária e Veterinária, Av. da República, 2780-157, Oeiras, Portugal

^3^Integrative biology platform, Environmental Research and Technology Platform, Luxembourg Institute of Science and Technology, Belvaux, Luxembourg

^4^Department of Chemical Engineering and Analytical Chemistry, University of Barcelona, Martí i Franquès 1-11, E-08028 Barcelona, Spain

^5^Serra Hunter Fellow, Generalitat de Catalunya, Spain

^6^Laboratory of Environmental Microbiology, Institute of Microbiology of the Czech Academy of Sciences, Videnska 1083, 14220 Praha 4, Czech Republic

^7^University of the West of Scotland, Paisley Campus, PA1 2BE Paisley, UK

^*^corresponding author ([spereira@itqb.unl.pt](mailto:spereira@itqb.unl.pt))

**Supplementary Information**

**Half maximal Effective Concentration of PCP against the metacommunity of fungi**


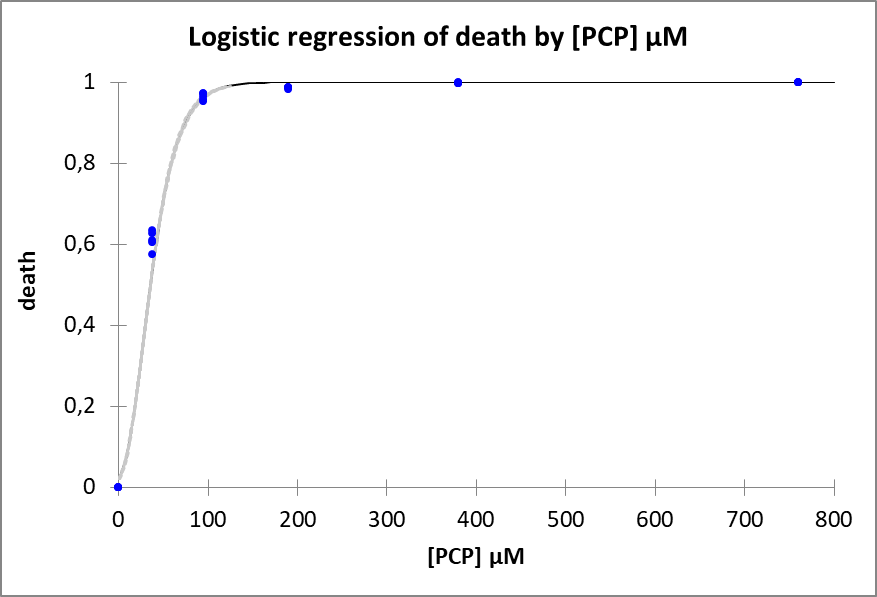

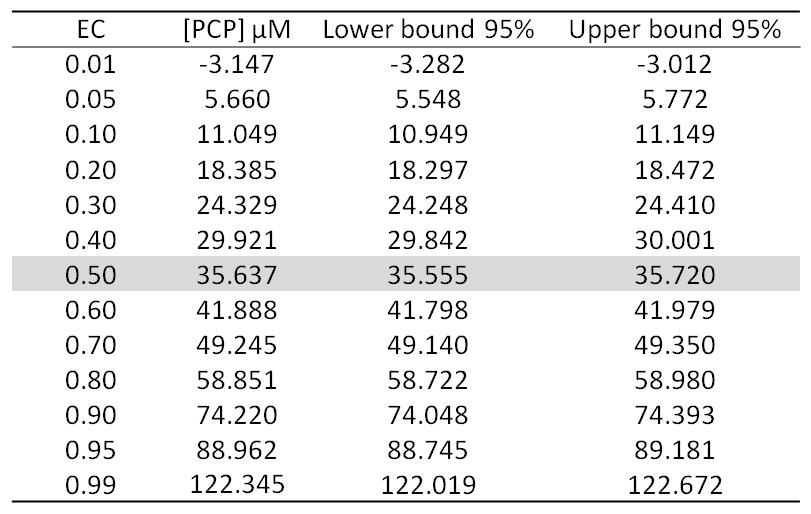


a

b

**Fig S1.** Half maximal Effective Concentration of PCP against the metacommunity of fungi. The logistic regression of death (CFU counts) in community-based cultures exposed to PCP, fitted using Gompertz distribution is displayed (a). The model prediction for the toxicity levels of the chemical is shown (b). The defined EC_50_ value was estimated as 35.637 µM, accordingly for simplicity in further experiments 38 µM PCP (= 10 mg.L^-1^) was used.

**Analysis of PCP degradation derivatives in the metacommunity of fungi along the cultivation time**

**Table S1.** PCP-derived metabolites identified in both mycelial and extracellular culture fractions, as well as in the abiotic controls. Full information is available in the Supplementary dataset 1.

| **Compound** | **Mycelia** | | | | **Extracellular** | | | | **Abiotic Control** | | |
| --- | --- | --- | --- | --- | --- | --- | --- | --- | --- | --- | --- |
|  | **3 days** | **5 days** | **7 days** | **10 days** | **3 days** | **5 days** | **7 days** | **10 days** | **1** | **2** | **3** |
| Trihydroxybenzene |  |  |  |  | ✓ | ✓ | ✓ | ✓ |  |  |  |
| Maleylacetate |  | ✓ | ✓ |  |  |  | ✓ | ✓ |  |  |  |
| 3-oxoadipate |  | ✓ |  |  |  |  | ✓ | ✓ |  |  |  |
| Tetrachlorohydroquinone |  | ✓ | ✓ |  | ✓ |  |  |  |  |  |  |
| Tetrachlororesorcinol |  |  |  |  | ✓ |  |  |  |  |  |  |
| Tetrachlorocathecol | ✓ | ✓ | ✓ | ✓ | ✓ |  |  |  | ✓ | ✓ | ✓ |
| Tetrachlorophenol | ✓ |  |  |  | ✓ |  |  |  | ✓ | ✓ | ✓ |
| Pentachlorophenol | ✓ | ✓ | ✓ | ✓ | ✓ | ✓ | ✓ | ✓ | ✓ | ✓ | ✓ |

**Profile of Utilization of carbon- and nitrogen-containing sources in the metacommunity along PCP exposure**

**Table S2.** Utilisation profile of substrates by the metacommunity along the PCP exposure time, displaying the most increased (left) and decreased (right) sources after 3 and 10 days compared to controls. Full list is available in the Supplementary dataset 2.

|  | **Functional category** | **C/N source** | **increase (%)** | **C/N source** | **decrease (%)** |
| --- | --- | --- | --- | --- | --- |
| **3 days** | Amines/Amides | 2-Amino Ethanol | 36.73 | Glucuronamide | 52.24 |
|  | Aminoacids | Glycyl-L-Glutamic acid | 32.30 |  |  |
|  | Carbohydrates | Maltitol | 45.95 | D-Arabinose | 72.27 |
|  |  | L-Sorbose | 37.20 | L-Fucose | 70.49 |
|  |  |  |  | N-Acetyl-D-Galactosamine | 70.44 |
|  |  |  |  | Lactulose | 69.35 |
|  |  |  |  | α-D-Lactose | 63.68 |
|  |  |  |  | Sedoheptulosan | 54.45 |
|  |  |  |  | β-Methyl-D-Galactoside | 48.62 |
|  | Carboxylic acid | N-Acetyl-L glutamic acid | 55.02 | D-Galacturonic acid | 85.21 |
|  | Miscellaneous | Adenosine-5'Monophosphate | 80.39 | D-Lactic Acid Methyl Ester | 46.42 |
|  |  | Uridine | 80.17 |  |  |
|  |  | Bromosuccinic acid | 41.34 |  |  |
|  |  | Salicin | 38.25 |  |  |
|  | Polymers |  |  | β-Cyclodextrin | 100.00 |
| **10 days** | Amines/Amides | Alaninamide | 56.74 | Glucuronamide | 100.00 |
|  |  |  |  | D-Glucosamine | 42.90 |
|  | Aminoacids | L-Phenylalanine | 45.14 |  |  |
|  | Carbohydrates | N-Acetyl-D-Galactosamine | 62.37 | N-Acetyl-D Mannosamine | 100.00 |
|  |  | D-Xylose | 30.69 | D-Tagatose | 94.94 |
|  |  |  |  | L-Arabinose | 89.68 |
|  |  |  |  | Lactulose | 81.65 |
|  |  |  |  | D-Arabinose | 70.53 |
|  |  |  |  | Sedoheptulosan | 53.19 |
|  |  |  |  | α-D-Lactose | 47.48 |
|  |  |  |  | L-Fucose | 45.22 |
|  |  |  |  | m-Inositol | 41.48 |
|  |  |  |  | D-Cellobiose | 37.00 |
|  |  |  |  | α-Methyl-D-Glucoside | 36.89 |
|  | Carboxylic acid | D-Glucuronic Acid | 36.18 | D-Galacturonic acid | 80.06 |
|  |  | N-Acetyl-L glutamic acid | 34.25 | γ-Hydroxy-butyric acid | 44.32 |
|  | Miscellaneous | Adenosine | 86.13 | Succinic Acid Mono-Methyl Ester | 44.09 |
|  |  | Uridine | 62.40 |  |  |
|  |  | Bromosuccinic acid | 36.09 |  |  |
|  | Polymers |  |  | α-Cyclodextrin | 100.00 |

**Analysis of the metacommunity composition and of the major players in PCP degradation**

**Table S3.** Results of the DESeq2 analysis (R based package) regarding the differential abundance (log_2_FC) of OTU’s at the third and the tenth day of exposure to PCP compared to controls, using raw and normalized data. To identify the OTU’s able to assimilate ^13^C PCP we have run comparisons of the controls with either the light or heavy DNA fractions, and between both light and heavy DNA fractions at two time points, as well as of each fraction between time points. No differential abundant OTU’s were attained between the light and heavy DNA fractions, possibly because ^13^C was not homogeneously assimilated by all the fungal cells.

|  |  | **Control vs. Light 3 days** | | **Control vs Heavy 3 days** | | **Control vs. Light 10 days** | | **Control vs Heavy 10 days** | | **Light 3 days vs Light 10 days** | | **Heavy 3 days vs Heavy 10 days** | |
| --- | --- | --- | --- | --- | --- | --- | --- | --- | --- | --- | --- | --- | --- |
|  |  | log_2_FC | *p-*value | log_2_FC | *p-*value | log_2_FC | *p-*value | log_2_FC | *p-*value | log_2_FC | *p-*value | log_2_FC | *p-*value |
| **Normalized Counts** | CL0003 |  |  |  |  |  |  |  |  |  |  | 1.54 | 2E-02 |
|  | CL0004 |  |  | 2.79 | 5E-02 |  |  |  |  |  |  |  |  |
|  | CL0006 |  |  |  |  | 3.34 | 4E-02 | 3.21 | 5E-02 | -3.31 | 4E-04 | -3.48 | 8E-07 |
|  | CL0007 |  |  |  |  |  |  |  |  | -2.98 | 6E-03 | -2.32 | 7E-03 |
|  | CL0008 |  |  |  |  | -7.67 | 5E-05 | -7.51 | 1E-04 | 9.73 | 2E-12 | 9.39 | 3E-15 |
|  | CL0009 | -2.40 | 4E-02 | -2.13 | 9E-03 |  |  |  |  |  |  |  |  |
|  | CL0010 |  |  |  |  |  |  |  |  | -4.36 | 2E-04 | -4.10 | 3E-04 |
|  | CL0011 |  |  |  |  |  |  |  |  | 4.95 | 5E-06 | 4.31 | 5E-05 |
|  | CL0012 | -10.44 | 3E-09 | -10.07 | 1E-09 |  |  |  |  |  |  |  |  |
|  | CL0015 | -7.08 | 4E-05 | -6.15 | 1E-04 |  |  |  |  |  |  |  |  |
|  | CL0020 |  |  |  |  |  |  | -8.56 | 5E-05 |  |  |  |  |

**Table S4.** The relative abundances of sequences of the OTUs able to assimilate ^13^C-labelled PCP (counts normalized and set to integers); OTUs with relative abundance above 100 counts, are listed. The 17 OTU’s presenting over 1,000 normalized counts (bolded) constitute over 97% of the total abundance of the metacommunity exposed to PCP. The abundance variations in the heavy DNA fraction at the third and the tenth day of exposure were used to classify the OTU’s regarding their timing in the assimilation of ^13^C PCP: “early” assimilators, OTU’s that present high abundance in the heavy fraction at early incubation with PCP, decreasing at the tenth day; “late” assimilators, OTU’s that are very abundant in the heavy fraction at later incubation with PCP, but are less abundant at the third day; and “steady” assimilators, OTU’s that are very abundant in the heavy fraction at both incubation times. Full information on the OTU’s, raw counts and the list of all OTU’s with abundance above 100 normalized counts (assimilators and non assimilators) is available in Supplementary dataset 3.

| **OTU** | **ID** | **Similarity (%) NCBI** | **Control 3 days** | **PCP Light 3 days** | **PCP Heavy 3 days** | **Control 10 days** | **PCP Light 10 days** | **PCP Heavy 10 days** | **Assimilation category** |
| --- | --- | --- | --- | --- | --- | --- | --- | --- | --- |
| **CL0001** | **KY037850_*Vanrija albida*** | 100 | 23085 | 19199 | 18628 | 28140 | 15016 | 13731 | **steady assimilator** |
| **CL0002** | **DTO 099-G3_*Fusarium oxysporum* species complex** | 100 | 9818 | 7749 | 7353 | 26369 | 16012 | 16546 | **late assimilator** |
| **CL0003** | **MG725820_*Trichoderma* sp.** | 100 | 7186 | 27317 | 20927 | 9831 | 7115 | 7247 | **early assimilator** |
| **CL0004** | **KY104335_*Naganishia randhawae*** | 99.5 | 4059 | 14712 | 19972 | 981 | 9267 | 10071 | **steady assimilator** |
| **CL0005** | **DTO 099-F7_*Penicillium vagum*** | 100 | 5439 | 13506 | 10962 | 1966 | 11727 | 10483 | **steady assimilator** |
| **CL0006** | **HG008760_*Trichoderma* sp.** | 100 | 1619 | 1833 | 1556 | 1576 | 19807 | 18834 | **late assimilator** |
| **CL0007** | **KY037851_*Solicoccozyma phenolicus*** | 100 | 9468 | 807 | 1500 | 4188 | 7264 | 7483 | **late assimilator** |
| **CL0008** | **KX302067_*Trichosporon* sp.** | 100 | 5059 | 6562 | 8034 | 4695 | 5 | 12 | **early assimilator** |
| **CL0009** | **AB737864_*Trichoderma hamatum*** | 100 | 9599 | 1072 | 1418 | 4355 | 1237 | 1876 | **steady assimilator** |
| **CL0010** | **KU987904_*Acremonium* sp.** | 99.4 | 481 | 197 | 246 | 1362 | 4051 | 5560 | **late assimilator** |
| **CL0011** | **KY320605_*Saitozyma podzolica*** | 100 | 3713 | 3140 | 4945 | 2248 | 107 | 200 | **early assimilator** |
| **CL0013** | **MG711821_*incertae sedis*** | 100 | 625 | 737 | 602 | 376 | 2422 | 1788 | **late assimilator** |
| **CL0014** | **KY315586_*Talaromyces verruculosus*** | 100 | 1 | 9 | 4 | 2 | 1783 | 2422 | **late assimilator** |
| **CL0016** | **DTO 099-E4_*Penicillium restrictum* species complex** | 100 | 374 | 821 | 1000 | 191 | 788 | 621 | **steady assimilator** |
| **CL0021** | **KY101694_*Apiotrichum vadense*** | 100 | 68 | 31 | 58 | 20 | 661 | 668 | **late assimilator** |
| **CL0022** | **KY687957_*Trichoderma linzhiense*** | 100 | 178 | 108 | 135 | 110 | 323 | 433 | **late assimilator** |
| **CL0024** | **DTO 100-A6_*Penicillium radiatolobatum*** | 100 | 222 | 324 | 467 | 130 | 95 | 83 | **early assimilator** |
| CL0026 | KY687942_*Trichoderma hirsutum* | 99.4 | 51 | 62 | 80 | 11 | 273 | 231 | late assimilator |
| CL0029 | KJ542213_*Thelonectria* sp. | 100 | 47 | 252 | 383 | 54 | 0 | 1 | early assimilator |
| CL0030 | KX100356_*Fusarium* sp. | 100 | 311 | 119 | 132 | 164 | 15 | 25 | early assimilator |
| CL0031 | KY105430_*Solicoccozyma aeria* | 100 | 163 | 141 | 226 | 230 | 1 | 1 | early assimilator |
| CL0032 | AF414294_*Trichoderma hamatum* | 95.9 | 5 | 12 | 18 | 2 | 223 | 225 | late assimilator |
| CL0034 | NR_152959_*Mucor moelleri f. californiensis* | 100 | 0 | 3 | 6 | 13 | 214 | 51 | late assimilator |
| CL0035 | MG193751_*Trichoderma pubescens* | 97.1 | 106 | 27 | 26 | 38 | 55 | 75 | late assimilator |
| CL0036 | DTO 099-F5_*Aspergillus tubingensis* | 100 | 95 | 51 | 29 | 22 | 51 | 66 | steady assimilator |
| **OTU** | **ID** |  | **Control 3 days** | **PCP Light 3 days** | **PCP Heavy 3 days** | **Control 10 days** | **PCP Light 10 days** | **PCP Heavy 10 days** | **Assimilation category** |
| CL0038 | AB986458_*Oidiodendron* sp. | 97.2 | 0 | 0 | 0 | 0 | 49 | 175 | late assimilator |
| CL0043 | *incertae sedis* |  | 1 | 3 | 20 | 0 | 88 | 87 | late assimilator |
| CL0046 | *incertae sedis*_2 |  | 1 | 1 | 4 | 0 | 100 | 85 | late assimilator |
| CL0050 | LC177644_*Sagenomella striatispora* | 100 | 1 | 5 | 4 | 2 | 78 | 72 | late assimilator |
| CL0052 | LC177652_*Sagenomella verticillata* | 100 | 68 | 73 | 75 | 18 | 0 | 0 | early assimilator |
| CL0054 | DTO 099-G3_1_*Fusarium oxysporum* species complex | 100 | 38 | 36 | 50 | 9 | 2 | 1 | early assimilator |
| CL0055 | MG722763_*Ustilago kamerunensis* | 96.7 | 81 | 53 | 15 | 38 | 5 | 11 | steady assimilator |
| CL0058 | KY687942_1_*Trichoderma hirsutum* | 99.4 | 28 | 35 | 45 | 2 | 19 | 15 | steady assimilator |
| CL0059 | KY101666_*Apiotrichum dulcitum* | 100 | 0 | 0 | 1 | 2 | 53 | 52 | late assimilator |
| CL0064 | JX030261_*Tricholomataceae* sp. | 99 | 22 | 7 | 17 | 38 | 19 | 15 | steady assimilator |
| CL0065 | *incertae sedis*_3 |  | 17 | 68 | 34 | 15 | 0 | 0 | early assimilator |
| CL0067 | LK052843_*incertae sedis* | 98.6 | 26 | 12 | 23 | 2 | 20 | 20 | steady assimilator |
| CL0068 | KX302022_*Trichosporon akiyoshidainum* | 100 | 9 | 14 | 15 | 15 | 29 | 19 | steady assimilator |
| CL0069 | DTO 099-E1_*Penicillium murcianum* | 100 | 1 | 22 | 42 | 2 | 30 | 6 | early assimilator |
| CL0071 | KX911872_*Gongronella butleri* | 99.4 | 85 | 7 | 23 | 6 | 0 | 0 | early assimilator |
| CL0073 | FJ265759_*Mycosphaerella* sp. | 87.6 | 19 | 14 | 15 | 36 | 11 | 13 | steady assimilator |

**Mycelial proteins of the metacommunity of fungi along PCP exposure**

**Table S5.** Mycelial proteins with major fold changes in abundance (log_2_FC) after PCP exposure for three and ten days compared to controls. Full data of the identified proteins is available in Supplementary dataset 4 and all fold changes are available in Supplementary dataset 5.

|  | **Functional category** | **3 days** | | | **10 days** | | |
| --- | --- | --- | --- | --- | --- | --- | --- |
|  |  | **Accession** | **Protein name** | **log_2_FC** | **Accession** | **Protein name** | **log_2_FC** |
| **Up-regulated** | *Aminoacid metabolism* | Q12650 | Sulfate adenylyltransferase | **7.16** | P05694 | 5-methyltetrahydropteroyltriglutamate homocysteine methyltransferase | **6.43** |
|  |  |  |  |  | Q9HFR6 | NADP-specific glutamate dehydrogenase | **6.57** |
|  | *ATP metabolic process* | P22068 | ATP synthase subunit β | **9.36** | P07038 | Plasma membrane ATPase | **8.45** |
|  |  | P85446 | ATP synthase subunit β | **9.06** | P28876 | Plasma membrane ATPase 2 | **7.49** |
|  | *Carbohydrate metabolic process* | P41751 | Aldehyde dehydrogenase | **7.42** | Q00640 | Glyceraldehyde-3-phosphate dehydrogenase | **8.22** |
|  |  | Q12560 | Enolase | **7.89** | P32636 | Glyceraldehyde-3-phosphate dehydrogenase 2 | **7.10** |
|  | *Mitochondrial functioning* | C8VG90 | Aconitate hydratase | **7.25** | P24487 | ATP synthase subunit alpha, mitochondrial | **8.45** |
|  |  |  |  |  | P51044 | Citrate synthase, mitochondrial | **7.49** |
|  | *Protein biosynthesis* | Q00251 | Elongation factor 1-alpha | **7.84** | P34825 | Elongation factor 1-α | **8.30** |
|  |  |  |  |  | Q9Y713 | Elongation factor 1-α | **7.10** |
|  | *Regulation, translation and signaling* | B6H2I7 | 40S ribosomal protein S0 | **7.57** | Q6FR65 | GTP-binding nuclear protein GSP1/Ran | **7.01** |
|  |  |  |  |  | C7YTD6 | 40S ribosomal protein S1 | **7.19** |
|  | *Stress response* | P08843 | Alcohol dehydrogenase | **8.50** | Q5B2V1 | Heat shock 70 kDa protein | **9.20** |
|  |  | G5EAZ2 | Thiamine thiazole synthase | **7.25** | P41797 | Heat shock protein SSA1 | **7.62** |
|  |  |  |  |  | C7Z8P6 | Thiamine thiazole synthase | **7.49** |
| **Down-regulated** | *Aminoacid metabolism* |  |  |  | P00369 | NADP-specific glutamate dehydrogenase | **-7.01** |
|  | *Carbohydrate metabolic process* | Q7RV85 | Enolase | **-8.55** | Q7RV85 | Enolase | **-9.67** |
|  |  | Q6W3C0 | Enolase | **-8.15** | P54118 | Glyceraldehyde-3-phosphate dehydrogenase | **-8.44** |
|  |  | Q00640 | Glyceraldehyde-3-phosphate dehydrogenase | **-8.58** | P87197 | Glyceraldehyde-3-phosphate dehydrogenase | **-7.92** |
|  |  | P35143 | Glyceraldehyde-3-phosphate dehydrogenase | **-8.15** | Q9HGY7 | Glyceraldehyde-3-phosphate dehydrogenase | **-7.73** |
|  |  | P41756 | Phosphoglycerate kinase | **-7.44** | P14228 | Phosphoglycerate kinase | **-7.88** |
|  |  | P31865 | Pyruvate kinase | **-8.04** |  |  |  |
|  | *Cytoskeleton* | Q6TCF2 | Actin | **-8.92** |  |  |  |
|  | *Mitochondrial functioning* | P24487 | ATP synthase subunit alpha, mitochondrial | **-8.15** | O00098 | Citrate synthase, mitochondrial | **-7.10** |
|  | *Protein biosynthesis* |  |  |  | A4QVP2 | ATP-dependent RNA helicase eIF4A | **-7.51** |
|  |  |  |  |  | P23301 | Eukaryotic translation initiation factor 5A-1 | **-7.10** |
|  | *Regulation, translation and signaling* | B0XWG9 | 40S ribosomal protein S0 | **-7.32** | Q01291 | 40S ribosomal protein S0 | **-7.83** |
|  | *Stress response* | P78695 | 78 kDa glucose-regulated protein homolog | **-7.75** | J9N5G7 | Thiamine thiazole synthase | **-8.35** |
|  |  | P41797 | Heat shock protein SSA1 | **-7.70** | P23617 | Thiamine thiazole synthase | **-7.68** |

**Extracellular proteins of the metacommunity of fungi along PCP exposure**

**Table S6.** Extracellular proteins that showed altered levels following PCP exposure for three (top, light grey) and ten (bottom, darker grey) days. The log_2_FC is displayed, as well as full information on the proteins. Entries that have been previously associated with pathogenesis, virulence or allergenic activity are marked with an *. Full data is available in Supplementary dataset 4.

|  | **Accession** | **log_2_FC** | **Order** | **Protein** | **Function** | **SignalP** | **FSKB** |
| --- | --- | --- | --- | --- | --- | --- | --- |
| 3 days | P17729* | 8.19 | Hypocreales | Glyceraldehyde-3-phosphate dehydrogenase 1 | Carbohydrate metabolic process | No | Yes |
|  | P35049* | 7.95 | Hypocreales | Trypsin | Aminoacid metabolism | No | Yes |
|  | Q96VJ7 | 7.88 | Hypocreales | NADP-specific glutamate dehydrogenase | Aminoacid metabolism | No | Yes |
|  | A4QUT2* | 7.22 | Magnaporthales | Catalase-peroxidase 2 | Stress response | Yes | Yes |
|  | Q03420* | 7.22 | Sordariomycetes | Alkaline proteinase | Antagonistic or mycoparasitic activity | Yes | Yes |
|  | A6N6J0* | 7.17 | Hypocreales | Endochitinase 46 | Antagonistic or mycoparasitic activity | Yes | Yes |
|  | B0XXF8* | 7.05 | Eurotiales | Glucan endo-1,3-beta-glucosidase | Sporulation | Yes | Yes |
|  | P17730* | 6.85 | Hypocreales | Glyceraldehyde-3-phosphate dehydrogenase 2 | Carbohydrate metabolic process | No | Yes |
|  | C8VG90 | 6.62 | Eurotiales | Aconitate hydratase | Carbohydrate metabolic process | No | No |
|  | Q4WP12 | 6.34 | Eurotiales | Peptidyl-prolyl cis-trans isomerase B | Protein biosynthesis | No | Yes |
| 10 days | J9MJK9 | 8.58 | Hypocreales | Transaldolase | Carbohydrate metabolic process | No | No |
|  | B0XXF8* | 7.32 | Eurotiales | Probable glucan endo-1,3-beta-glucosidase | Sporulation | Yes | Yes |
|  | Q7S986 | 7.32 | Sordariales | Glucose-6-phosphate isomerase | Carbohydrate metabolic process | No | Yes |
|  | P42882 | 6.71 | Eurotiales | 4-amino-5-hydroxymethyl-2-methylpyrimidine phosphate synthase | Aminoacid metabolism | No | Yes |
|  | Q00616* | 6.33 | Hypocreales | Cytochrome P450 55A2 | Stress response | No | Yes |
|  | Q03420* | 6.33 | Hypocreales | Alkaline proteinase | Antagonistic or mycoparasitic activity | Yes | Yes |
|  | P17729* | 6.18 | Hypocreales | Glyceraldehyde-3-phosphate dehydrogenase 1 | Carbohydrate metabolic process | No | Yes |
|  | A6N6J0* | 5.82 | Hypocreales | Endochitinase 46 | Antagonistic or mycoparasitic activity | Yes | Yes |
|  | A4QUT2* | -8.47 | Magnaporthales | Catalase-peroxidase 2 | Stress response | Yes | Yes |
|  | Q4WLN1 | -8.47 | Eurotiales | Aconitate hydratase | Carbohydrate metabolic process | No | Yes |
|  | Q9HGU3 | -8.33 | Sordariales | NADP-specific glutamate dehydrogenase | Aminoacid metabolism | No | Yes |
|  | Q9HDT3 | -7.60 | Pleosporales | Enolase | Carbohydrate metabolic process | No | Yes |
|  | O13639 | -7.02 | Schizosaccharomycetales | Adenosylhomocysteinase | Aminoacid metabolism | No | Yes |
|  | Q8TFJ2 | -7.02 | Agaricales | Glyceraldehyde-3-phosphate dehydrogenase | Carbohydrate metabolic process | No | Yes |
|  | Q10318 | -6.61 | Schizosaccharomycetales | Putative dihydroxy-acid dehydratase | Aminoacid metabolism | No | Yes |

**Complementary assays to assess pathogenic potential**

*

**Fig S2.** The pH of the media of the metacommunity-based cultures exposed or unexposed to PCP was measured at the start and at the tenth day of incubation. The pH of the medium at the last incubation time point increased significantly (marked with *) only in the metacommunity cultures exposed to PCP.

**Fig S3.** Metabolic activity of metacommunity-based cultures exposed, or unexposed, to PCP during ten days, in the presence of miconazole (acute effect). The antifungal compound significantly reduced the metabolic activity of control cultures to a minimum, but not of the metacommunity exposed to PCP (the range of absorbance values to metabolic active cells is 0.2 – 0.7).

*Experimental details*: Metacommunity-based cultures grown for ten days in medium with 38 µM of PCP, or in a control medium, were exposed or not to 0.1 mg/mL miconazole for 24h (30°C, 90 rpm). At the end of the acute treatment with the antifungal compound, the metabolic activity of the cultures was estimated using the MTT (3-(4,5-dimethylthiazol-2-yl)-2,5-diphenyltetrazolium bromide) tetrazolium reduction assay. Briefly, the cultures were treated with 1/10 volume of 5 mg.mL^-1^ MTT in 1% NaCl (4h, 30°C, 90 rpm), then after removal of the medium, an equal volume of 0.1M HCl in isopropanol was added (4h, 30°C, 90 rpm). The supernatants’ absorbance measured at 570 nm and 630 nm to remove noise in the background; Student’s *t*-test and Kruskal-Wallis *H* test were used to infer (and confirm) significant differences between conditions (marked with different letters).

**Table S7 –** Characterisation of sampling sites and soil samples, including descriptors for the cork oak forest locations, physicochemical characterisation of the soils (namely C/N), PCP levels and fungal CFUs.

| **Location** | **Coordinates (GPS)** | | **Vegetation** | **Litter** | **Humidity (%)** | **pH** | **Organic Matter (%)** | **C/N** | **[PCP] (µg.Kg^-1^)** | **Fungal CFU's (colonies.g soil^-1^)** |
| --- | --- | --- | --- | --- | --- | --- | --- | --- | --- | --- |
|  | **Easting** | **Northing** |  |  |  |  |  |  |  |  |
| AH1 | 008°51’52.00“ | 36°46’47.50“ | sparse undergrowth (high bushes) | leaf litter | 23.5 | 5.4 | 5.8 | 15.8 | 28.8 | 1.3E+04 |
| AH2 | 008°51’53.80“ | 36°46’49.20“ | Abundant undergrowth (high bushes) | dense layers of leaf litter | 26.9 | 5.6 | 7.6 | 23.2 | 13.2 | 3.4E+04 |
| AH3 | 008°51’52.10“ | 36°46’50.40“ | sparse undergrowth | dense layers of leaf litter | 26.0 | 5.0 | 8.2 | 53.8 | 20.7 | 1.7E+04 |
